# Gynaecological Laparoscopy Surgery Complicated Into CO_2_ Embolism, Cardiac Arrest and Severe ARDS Requiring ECMO Therapy

**DOI:** 10.1002/rcr2.70387

**Published:** 2026-05-01

**Authors:** Jawdat Alali, Umm E. Amara, Malek Abusannuga, Umme Nashrah, Firdous Ummunnisa, Nissar Shaikh

**Affiliations:** ^1^ Surgical Intensive Care Unit, Hamad Medical Corporation (HMC) Doha Qatar; ^2^ Deccan College of Medical Sciences Hyderabad India; ^3^ Halima Al‐Tamimi OBGY Clinic Doha Qatar

**Keywords:** ARDS, cardiac arrest, CO_2_E, ECMO, EtCO_2_, gestational uterine fundus injury, hypoxia, laparoscopy

## Abstract

Laparoscopic surgeries have revolutionised surgical practice due to their advantages, including minimal invasiveness, fewer postoperative complications, quicker recovery and reduced hospital stay. Carbon dioxide (CO_2_) is used for abdominal insufflation because of its inert nature, colorlessness, low cost, high solubility, low combustibility and wide availability. Despite these advantages, laparoscopy is not without complications—it can cause hypotension, arrhythmia, pulmonary barotrauma and, rarely, carbon dioxide embolism (CO_2_E) and cardiac arrest. CO_2_E is commonly associated with cardiac arrest and has rarely been linked to acute respiratory distress syndrome (ARDS). Cases of CO_2_E complicated by ARDS have shown improvement with conventional invasive lung‐protective ventilation. However, CO_2_E complicated by severe ARDS with hypoxia refractory to standard therapy has not been reported. We report a case of CO_2_E complicated by severe ARDS with refractory hypoxia requiring extracorporeal membrane oxygenation (ECMO) therapy, which resulted in a favourable outcome.

## Graphical abstract

We report a case of CO_2_ embolism complicated by severe ARDS with refractory hypoxia requiring extracorporeal membrane oxygenation (ECMO) therapy, which resulted in a favourable outcome.

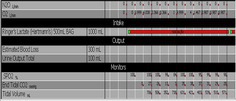

## 1 Introduction

Laparoscopic surgeries are increasingly performed due to their minimally invasive nature, reduced postoperative pain, early ambulation, and shorter hospital stays. To enhance visualisation and create adequate operative space, the abdomen is insufflated with gas—most commonly carbon dioxide—because of its favourable properties. Although generally safe, carbon dioxide can, in rare cases, lead to potentially fatal carbon dioxide embolism (CO_2_E) [1].

The incidence of CO_2_E varies by procedure. Bonjer et al., in a meta‐analysis of 489,335 laparoscopic surgeries, reported only seven cases, corresponding to an incidence of 0.0013% [2]. The highest occurrence has been described during laparoscopic total hysterectomy and endoscopic vein harvesting [3]. The main risk factor is vascular or solid organ injury during Veress needle insertion, allowing CO_2_ entry into the circulation. CO_2_E may cause hemodynamic collapse or cardiac arrest and has rarely been associated with acute respiratory distress syndrome (ARDS) [4].

ARDS secondary to CO_2_E with refractory hypoxemia unresponsive to conventional ventilation has not been previously reported. We describe a case of CO_2_E complicating gynecologic laparoscopy, progressing to severe ARDS requiring extracorporeal membrane oxygenation (ECMO) support, with complete recovery.

## 2 Case Report

A 28‐year‐old gravida 2, para 1 at 12 weeks of gestation presented to a private facility with persistent vomiting and lower abdominal pain. She was referred to our tertiary centre for further evaluation. Ultrasonography revealed findings consistent with right ovarian torsion, and emergency diagnostic laparoscopy with possible oophorectomy was planned. Her history included a previous ectopic pregnancy managed by right salpingectomy.

On admission, she was alert and hemodynamically stable. Abdominal examination showed mild right iliac fossa tenderness without peritonism. Pelvic ultrasound demonstrated an enlarged right ovary (6.6 × 4 cm) with absent intraovarian flow and sluggish peripheral perfusion, consistent with torsion. A viable intrauterine pregnancy was also noted. She was taken for laparoscopic intervention under general anaesthesia.

**FIGURE 1:**
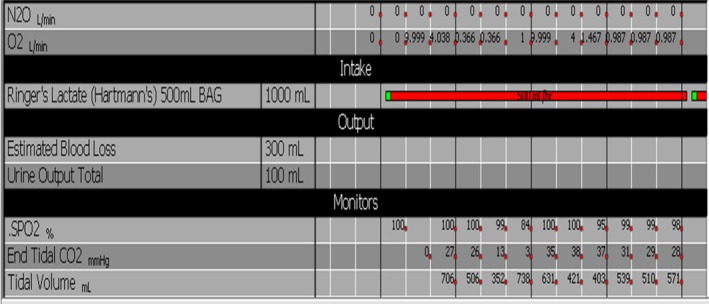
Showing an abrupt drop in end tidal carbon dioxide.

Anaesthesia was induced with propofol and fentanyl, followed by rocuronium. Endotracheal intubation was uneventful. Initial insufflation attempts were difficult, likely due to adhesions from prior salpingectomy and the torsed, congested ovary tethered to the abdominal wall, predisposing to preperitoneal or vascular injury during Veress needle insertion. Insufflation was achieved on the second attempt. Shortly afterward, a sudden decline in end‐tidal CO_2_ from 26 to 13 mmHg occurred, immediately followed by cardiac asystole (Figure 1).

Cardiopulmonary resuscitation (CPR) was initiated immediately, accompanied by rapid abdominal desufflation. Return of spontaneous circulation (ROSC) occurred after a single cycle of chest compressions. A phenylephrine infusion was started to maintain hemodynamic stability. Once stabilised, laparoscopy was resumed; however, aspiration of blood through the Veress needle prompted conversion to open laparotomy.

Exploratory laparotomy revealed approximately 500 mL of hemoperitoneum, a laceration at the uterine fundus, and right ovarian torsion. A right oophorectomy was performed, the uterine injury was repaired, and peritoneal lavage was completed. Postoperatively, the patient was awake, hemodynamically stable, and successfully extubated before transfer to the post‐anaesthesia care unit (PACU).

In the PACU, the patient developed progressive hypoxia and agitation, with oxygen desaturation to 80%. Within 15 min, she required endotracheal intubation and mechanical ventilation. About 200 mL of pink, frothy secretions was suctioned, and 40 mg of intravenous furosemide was administered. She was transferred to the intensive care unit (ICU) for further management.

In the ICU, she was sedated and paralysed, with norepinephrine infusion for hemodynamic support. Peak airway pressures were 34–36 cm H_2_O, and despite 100% FiO_2_, arterial oxygen saturation remained 92%. Her P/F ratio was 126, consistent with moderate to severe ARDS.

The patient remained tachycardic (120–130 bpm). Transthoracic echocardiography showed preserved left ventricular function (LVEF 54%) without wall motion abnormalities but elevated pulmonary artery systolic pressure (52 mmHg), suggesting pulmonary hypertension. ECG showed sinus tachycardia, and cardiac and inflammatory markers were normal.

By ICU day 1, she developed oliguria with rising creatinine and urea, consistent with acute kidney injury. Intravenous furosemide was administered, and vasopressin was added to norepinephrine for hemodynamic support under advanced monitoring.

**FIGURE 2:**
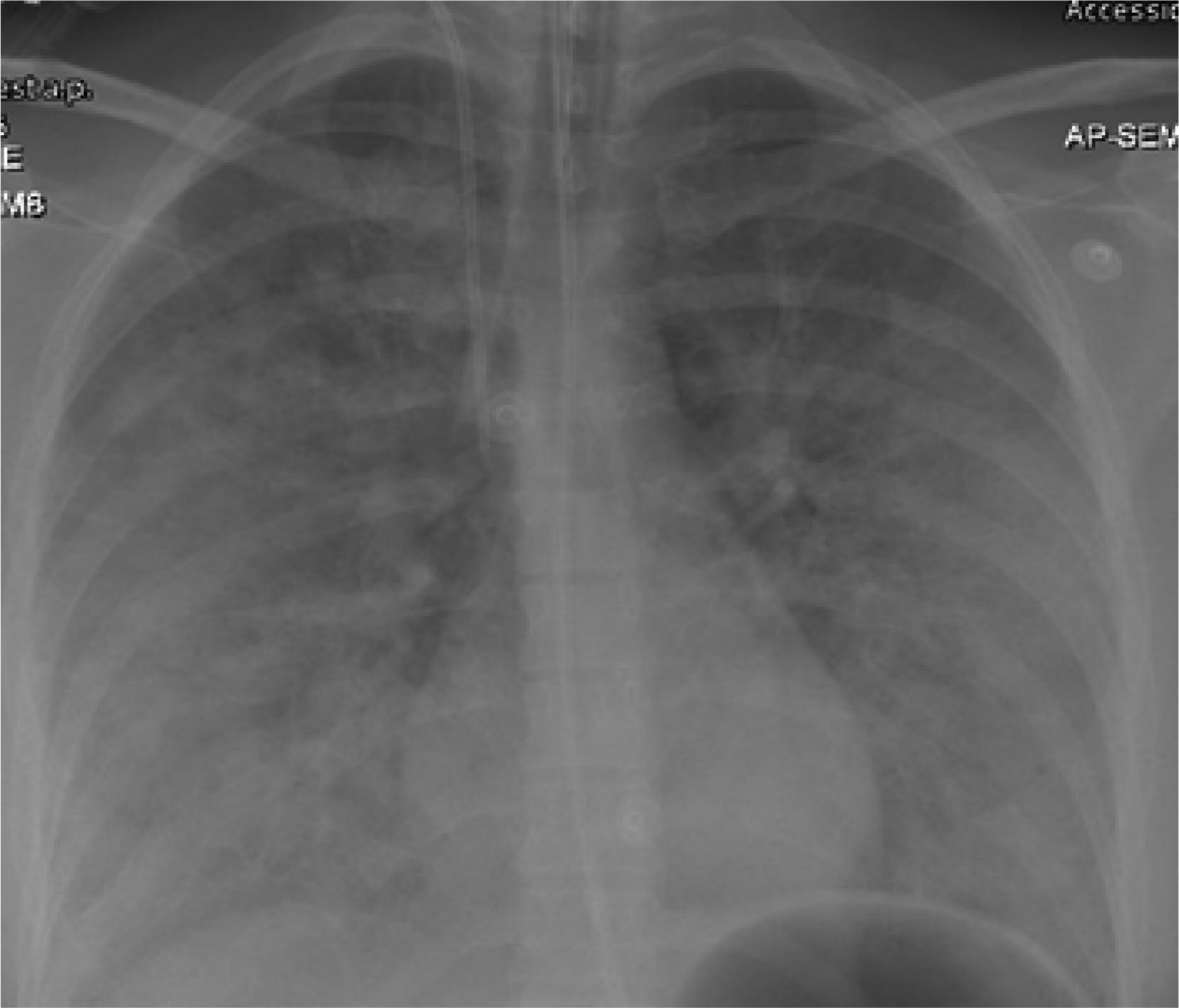
Showing diffuse bilateral lung infiltrates.

**FIGURE 3:**
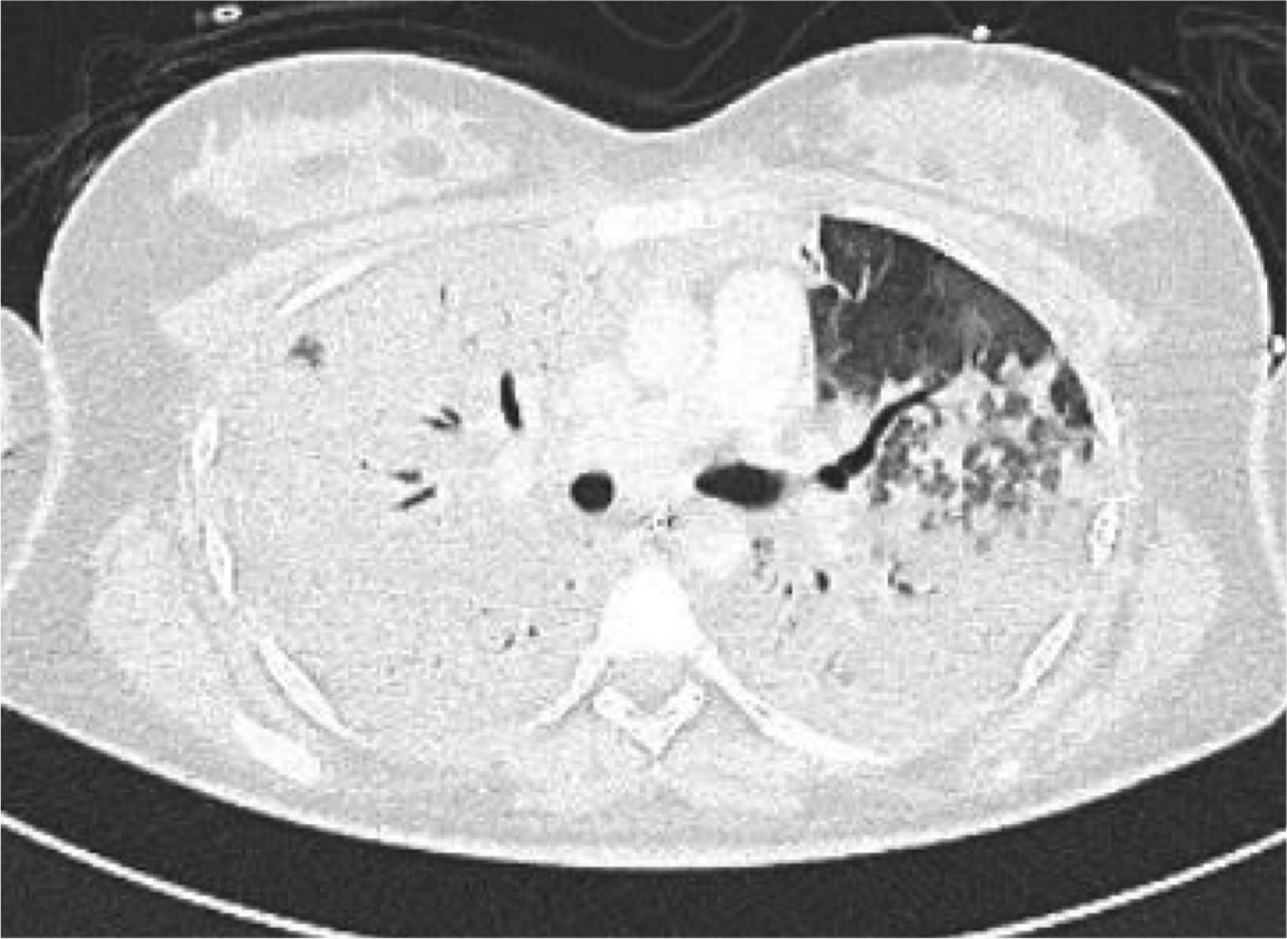
Axial Chest CT scan with IV contrast (lung window). There is diffuse, near‐complete parenchymal opacification of both lungs, with air bronchograms noted.

**FIGURE 4:**
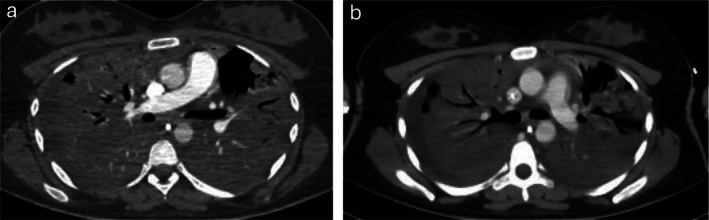
Axial Chest CT scan with contrast (mediastinal window). The pulmonary trunk, right and left main pulmonary arteries, and their lobar branches are well opacified, with no evidence of intraluminal filling defects.

Despite these measures, respiratory function worsened. Mechanical ventilation with 100% FiO_2_ and PEEP of 18 cm H_2_O maintained only 90% oxygen saturation. Serum lactate increased to 4.7 mmol/L, indicating poor tissue perfusion. Chest radiography showed bilateral diffuse infiltrates (Figure 2), and contrast‐enhanced CT demonstrated near‐complete parenchymal opacification with air bronchograms (Figure 3), consistent with acute respiratory distress syndrome (ARDS). Axial CT excluded pulmonary embolism (Figure 4). Oxygen saturation fell to 60%–70%, with peak and plateau pressures of 40–45 and 30–34 cm H_2_O, respectively. The P/F ratio was 46, confirming severe ARDS.

Fluid status was carefully managed to avoid overload, and the patient responded to conservative diuresis; continuous renal replacement therapy (CRRT) was not required. Taken together, the clinical course supports severe ARDS as the primary driver of hypoxemia rather than fluid overload.

**FIGURE 5:**
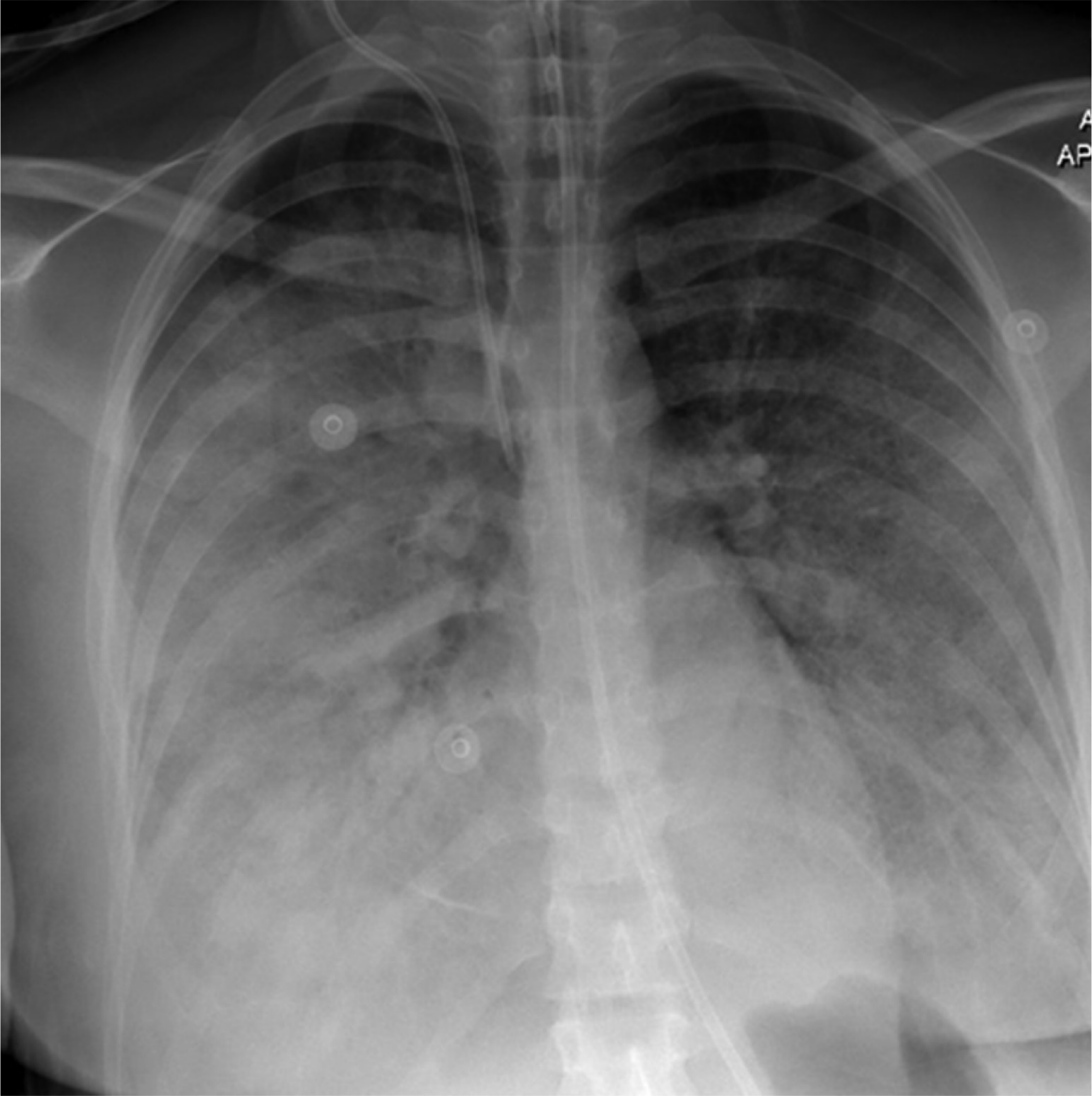
Showing diffuse bilateral lung infiltrates at the start of ECMO therapy.

The ECMO team was promptly activated, and venovenous (VV) ECMO was initiated via bilateral femoral cannulation for severe respiratory failure. ECMO settings were adjusted using serial arterial blood gases to optimise oxygenation and ventilation. By day 3, hemodynamic improvement allowed gradual weaning from vasopressors (Figure 5).

During ECMO, the patient's oxygenation and lung compliance progressively improved. Post‐cannulation, O_2_ saturation reached 100% on pressure control ventilation. ECMO weaning was initiated by gradually reducing sweep gas and adjusting ventilatory settings. By day 3, the sweep was briefly reduced to 0, with transient desaturation corrected by increasing FiO_2_. Vasopressors were fully discontinued by day 4, and sedation was tapered. Foetal heart tones were absent, and medical termination was performed with intravaginal misoprostol.

By day 6, she was awake, communicating, off sedation and vasopressors, tolerating oral intake, and passing urine adequately, allowing extubation and transition to high‐flow nasal cannula. ECMO was discontinued on day 7, and HFNC was stopped by day 9.

On day 10, she developed left lower limb pain; Doppler confirmed deep venous thrombosis, and anticoagulation with enoxaparin was started. She was transferred to the general ward on day 11 and discharged on day 13. Outpatient follow‐up at 3 and 6 months showed continued clinical stability.

## 3 Discussion

Laparoscopic surgery is widely used for elective and emergency abdominal and pelvic procedures due to advantages over open laparotomy [5]. However, it carries risks, including Veress needle or trocar injuries and carbon dioxide embolism (CO_2_E) [6].

Carbon dioxide (CO_2_) is preferred for insufflation as it is inert, colourless, inexpensive, readily available, highly soluble in blood, and has low combustibility [3]. CO_2_E is rare and varies by procedure. In gynaecological laparoscopy, 15 cases were reported among 113,253 procedures (0.013%) [7, 8]. In one study, intraoperative monitoring detected CO_2_ embolic signals in all laparoscopic total hysterectomy cases, particularly during transection of the round and broad ligaments; nonetheless, these embolic events were minor and hemodynamically insignificant [9]. Incidence may reach 17% in endoscopic vein harvesting [10, 11].

CO_2_E can result from vascular injury during Veress needle insertion, allowing gas to enter circulation. A sudden bolus may obstruct the pulmonary artery or right ventricle, potentially causing cardiac arrest. Blood aspiration through the needle should prompt concern, as in our patient [12], where concurrent uterine injury likely increased CO_2_ absorption.

Diagnosis can be made with transesophageal echocardiography (TEE), detecting emboli as small as 0.1 mL/kg, or pulmonary artery catheterization, detecting 0.5 mL/kg [13, 14]. Continuous end‐tidal CO_2_ (EtCO_2_) monitoring is a sensitive, non‐invasive tool [15]. CO_2_E may present as abrupt EtCO_2_ rises (~4%) or, more commonly, sudden decreases reflecting pulmonary obstruction. In our case, EtCO_2_ dropped abruptly (Figure 1) [3, 8].

Management involves immediate cessation of insufflation, abdominal desufflation, cardiopulmonary resuscitation and hemodynamic support. Ventilation with 100% oxygen aids CO_2_ elimination. Hyperbaric oxygen may benefit neurological involvement.

If CO_2_E progresses to acute respiratory distress syndrome (ARDS), lung‐protective ventilation with stepwise PEEP is indicated [3, 4]. Zhu et al. reported CO_2_ embolism after laparoscopic venous sinus injury progressing to ARDS, successfully managed with lung‐protective strategies [4].

Our case involved severe ARDS from CO_2_E, refractory to conventional ventilation, requiring veno‐venous extracorporeal membrane oxygenation (ECMO). The patient improved, was successfully extubated, and developed deep venous thrombosis without neurological deficits. This is the first reported laparoscopic CO_2_E case complicated by ARDS requiring ECMO. A prior case described CO_2_E causing cardiac arrest treated with ECMO but without ARDS [16]. Mortality for CO_2_E is ~28%, depending on embolized gas volume and initial compromise [17].

Preventive strategies include maintaining insufflation ≤ 12 mmHg [18], careful Veress needle insertion, and confirming placement by absence of blood aspiration. The Hasson open‐entry technique shows zero reported CO_2_E and may be safer in selected cases [2, 19].

In conclusion, carbon dioxide embolism (CO_2_E) is a rare but potentially fatal complication of laparoscopic surgery. It is most commonly associated with cardiovascular collapse and cardiac arrest. In severe cases, CO_2_E can lead to acute respiratory distress syndrome (ARDS) and refractory hypoxemia that is unresponsive to conventional therapies, necessitating extracorporeal membrane oxygenation (ECMO) support. In our patient, a history of prior pelvic surgery contributed to a technically challenging Veress needle insertion, resulting in gestational uterine fundal injury, which likely increased the risk of CO_2_E. Diagnosis was suggested by an abrupt decline in end‐tidal CO_2_ (EtCO_2_). ECMO therapy proved to be life‐saving in this case.

## Author Contributions

Jawdat Alali contributed to the conception of the work, data collection, drafting of the manuscript, and preparation of figures. Malek Abusannuga contributed to manuscript preparation and revision of the final draft. Umm E Amara and Umme Nashrah participated in the literature review and manuscript preparation. Firdous Ummunnisa contributed to the clinical management of the case and data interpretation. Nissar Shaikh supervised the work, provided critical revisions, and approved the final version of the manuscript. All authors read and approved the final manuscript.

## Consent

The authors declare that written informed consent was obtained for the publication of this manuscript and accompanying images and attest that the form used to obtain consent from the patient(s) complies with the Journal requirements as outlined in the author guidelines.

## Conflicts of Interest

The authors declare no conflicts of interest.

## Data Availability

The data that support the findings of this study are available from the corresponding author upon reasonable request.
